# Supplementary material for: Expansion of sandhill cranes (Grus canadensis) in east Asia during the non-breeding period
Source: PeerJ. 2019 Aug 30;7:e7545. doi: 10.7717/peerj.7545 (PMC6718156; doi:10.7717/peerj.7545)
Supplement: Supplemental Information 1 [file peerj-07-7545-s002.docx]

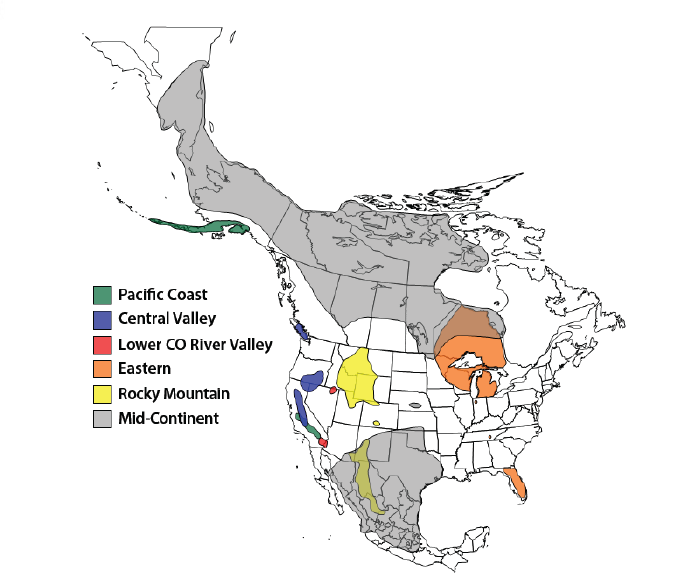


Figure A1. Approximate Nesting, Winter, and Primary Migration Staging Areas of the Six Migratory Sandhill Crane Populations (USFWS, 2018)


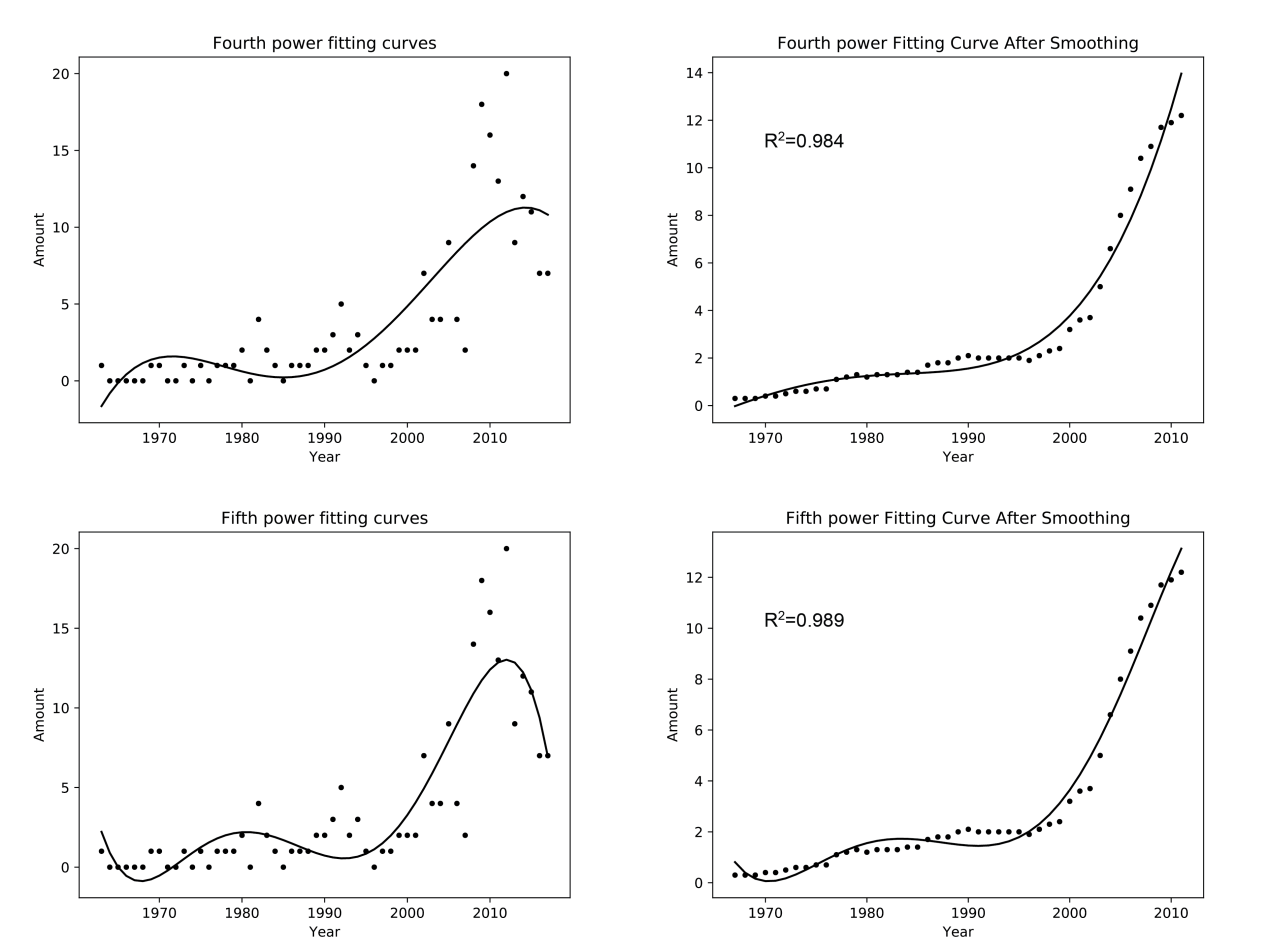


Figure A2. The scatter plot of the number of the sandhill crane in Asia in the past half century and its fourth and fifth fitting curve before and after doing simple Mean-value filter.

Table A1 Data source table

The location of sandhill cranes that appeared in East Asia during the last half a century and their corresponding reference

| Time | Location | Reference |
| --- | --- | --- |
| 1963-1964 | Izumi, Kagoshima, Japan | Crane Park Izumi, 2017 |
| 1969-1970 | Kushiro, Hokkaido, Japan | Masatomi, 1972 |
| 1970-1971 | Yancheng, Jiangsu, China | Wang, 2006 |
| 1973-1974 | Izumi, Kagoshima, Japan | Crane Park Izumi, 2017 |
| 1975-1976 | Izumi, Kagoshima, Japan | Crane Park Izumi, 2017 |
| 1977-1978 | Izumi, Kagoshima, Japan | Crane Park Izumi, 2017 |
| 1978-1979 | Shuyang, Jiangsu, China | Kuang *et al.*, 1981 |
| 1979-1980 | Izumi, Kagoshima, Japan | Crane Park Izumi, 2017 |
| 1980-1981 | Izumi, Kagoshima, Japan | Crane Park Izumi, 2017 |
| 1982-1983 | Izumi, Kagoshima, Japan | Crane Park Izumi, 2017 |
| 1983-1984 | Izumi, Kagoshima, Japan | Crane Park Izumi, 2017 |
| 1984-1985 | Poyang Lake, Jiangxi, China | Liu & Ding, 1988 |
| 1986-1987 | Poyang Lake, Jiangxi, China | Qian, 2005 |
| 1987-1988 | Izumi, Kagoshima, Japan | Crane Park Izumi, 2017 |
| 1988-1989 | Izumi, Kagoshima, Japan | Crane Park Izumi, 2017 |
| 1989-1990 | Izumi, Kagoshima, Japan | Crane Park Izumi, 2017 |
| 1990-1991 | Yellow River Delta Nature Reserve, Shandong, China | Wang, 2006 |
|  | Izumi, Kagoshima, Japan | Crane Park Izumi, 2017 |
| 1991-1992 | Izumi, Kagoshima, Japan | Crane Park Izumi, 2017 |
| 1992-1993 | Izumi, Kagoshima, Japan | Crane Park Izumi, 2017 |
| 1993-1994 | Izumi, Kagoshima, Japan | Crane Park Izumi, 2017 |
|  | Izumi, Kagoshima, Japan | GBIF, 2017 |
|  | Izumi, Kagoshima, Japan | GBIF, 2017 |
| 1994-1995 | Izumi, Kagoshima, Japan | Crane Park Izumi, 2017 |
| 1995-1996 | Izumi, Kagoshima, Japan | Crane Park Izumi, 2017 |
| 1997-1998 | Izumi, Kagoshima, Japan | Crane Park Izumi, 2017 |
| 1998-1999 | Izumi, Kagoshima, Japan | Crane Park Izumi, 2017 |
| 1999-2000 | Yellow River Wetland National Nature Reserve, Henan, China | Bird Talker, 2018 |
|  | Izumi, Kagoshima, Japan | Crane Park Izumi, 2017 |
|  | Izumi, Kagoshima, Japan | GBIF, 2017 |
| 2000-2001 | Izumi, Kagoshima, Japan | GBIF, 2017 |
|  | Izumi, Kagoshima, Japan | Crane Park Izumi, 2017 |
| 2001-2002 | Izumi, Kagoshima, Japan | Crane Park Izumi, 2017 |
|  | Izumi, Kagoshima, Japan | GBIF, 2017 |
| 2002-2003 | Izumi, Kagoshima, Japan | Crane Park Izumi, 2017 |
|  | Zhalong National Nature Reserve, Heilongjiang, China | Ma, 2003 |
| 2003-2004 | Poyang Lake, Jiangxi, China | Wang, 2006 |
|  | Izumi, Kagoshima, Japan | Crane Park Izumi, 2017 |
|  | Izumi, Kagoshima, Japan | GBIF, 2017 |
|  | Izumi, Kagoshima, Japan | GBIF, 2017 |
| 2004-2005 | Izumi, Kagoshima, Japan | GBIF, 2017 |
|  | Shanghai, China | Bird Talker, 2018 |
|  | Shanghai, China | Bird Talker, 2018 |
|  | Izumi, Kagoshima, Japan | Crane Park Izumi, 2017 |
|  | Izumi, Kagoshima, Japan | GBIF, 2017 |
| 2005-2006 | Chungcheongnam, Korea | GBIF, 2017 |
|  | Izumi, Kagoshima, Japan | Crane Park Izumi, 2017 |
|  | Chongming Dongtan Nature Reserve, Shanghai, China | Cai *et al.*, 2011 |
|  | Suncheon Bay Wetland, Suncheon, Jeonnam, Korea | Naver Blog, 2006 |
|  | Nanjing, Jiangsu, China | Bird Talker, 2018 |
|  | Yancheng National Wetland Nature Reserve for Rare Birds, Jiangsu, China | Bird Talker, 2018 |
|  | Yancheng National Wetland Nature Reserve for Rare Birds, Jiangsu, China | Bird Talker, 2018 |
|  | Chongming Dongtan Nature Reserve, Shanghai, China | Bosma, 2017 |
| 2006-2007 | Yellow River Delta Nature Reserve, Shandong, China | Wang *et al.*, 2007 |
|  | Izumi, Kagoshima, Japan | Crane Park Izumi, 2017 |
| 2007-2008 | Izumi, Kagoshima, Japan | Crane Park Izumi, 2017 |
|  | Izumi, Kagoshima, Japan | GBIF, 2017 |
|  | Izumi, Kagoshima, Japan | GBIF, 2017 |
|  | Izumi, Kagoshima, Japan | GBIF, 2017 |
| 2008-2009 | Izumi, Kagoshima, Japan | GBIF, 2017 |
|  | Zhalong National Nature Reserve, Heilongjiang, China | Cheng et al., 2014 |
|  | Izumi, Kagoshima, Japan | Crane Park Izumi, 2017 |
|  | Izumi, Kagoshima, Japan | GBIF, 2017 |
|  | Izumi, Kagoshima, Japan | GBIF, 2017 |
|  | Izumi, Kagoshima, Japan | GBIF, 2017 |
|  | Yancheng National Wetland Nature Reserve for Rare Birds, Jiangsu, China | This paper |
|  | Kagoshima, Japan | Bird Fan, 2014 |
| 2009-2010 | Xinqing Wetland, Yichun, Jilin, China | This paper |
|  | Yancheng National Wetland Nature Reserve for Rare Birds, Jiangsu, China | Bird Talker, 2018 |
|  | Yancheng National Wetland Nature Reserve for Rare Birds, Jiangsu, China | Bird Talker, 2018 |
|  | Izumi, Kagoshima, Japan | Crane Park Izumi, 2017 |
|  | Izumi, Kagoshima, Japan | GBIF, 2017 |
|  | Izumi, Kagoshima, Japan | GBIF, 2017 |
|  | Izumi, Kagoshima, Japan | GBIF, 2017 |
|  | Momoge National Nature Reserve, Jilin, China | Yu *et al.*, 2011 |
|  | Xinqing Wetland, Yichun, Jilin, China | Ifeng, 2010 |
|  | Yancheng National Wetland Nature Reserve for Rare Birds, Jiangsu, China | Bird Talker, 2018 |
|  | Yancheng National Wetland Nature Reserve for Rare Birds, Jiangsu, China | Bird Talker, 2018 |
|  | Yancheng National Wetland Nature Reserve for Rare Birds, Jiangsu, China | This paper |
|  | Shanghai, China | Bird Talker, 2018 |
|  | Shanghai, China | Bird Talker, 2018 |
|  | Kagoshima, Japan | Bird Fan, 2014 |
|  | Izumi, Kagoshima, Japan | This paper |
| 2010-2011 | Izumi, Kagoshima, Japan | GBIF, 2017 |
|  | Sheyang, Yancheng, Jiangsu, China | GBIF, 2017 |
|  | Sheyang, Yancheng, Jiangsu, China | GBIF, 2017 |
|  | Yancheng National Wetland Nature Reserve for Rare Birds, Jiangsu, China | Cheng et al., 2014 |
|  | Chongming Dongtan Nature Reserve, Shanghai, China | Cheng et al., 2014 |
|  | Yancheng National Wetland Nature Reserve for Rare Birds, Jiangsu, China | Bird Talker, 2018 |
|  | Chongming Dongtan Nature Reserve, Shanghai, China | Bird Talker, 2018 |
|  | Yancheng National Wetland Nature Reserve for Rare Birds, Jiangsu, China | Bird Talker, 2018 |
|  | Chongming Dongtan Nature Reserve, Shanghai, China | Bird Talker, 2018 |
|  | Zhengzhou, Henan, China | Bosma, 2017 |
|  | Izumi, Kagoshima, Japan | Crane Park Izumi, 2017 |
|  | Yancheng National Wetland Nature Reserve for Rare Birds, Jiangsu, China | Bird Talker, 2018 |
|  | Sheyang, Yancheng, Jiangsu, China | Bird Talker, 2018 |
| 2011-2012 | Chongming Dongtan Nature Reserve, Shanghai, China | GBIF, 2017 |
|  | Yancheng National Wetland Nature Reserve for Rare Birds, Jiangsu, China | Cheng et al., 2014 |
|  | Chongming Dongtan Nature Reserve, Shanghai, China | Cheng et al., 2014 |
|  | Momoge National Nature Reserve, Jilin, China | Cheng et al., 2014 |
|  | Zhalong National Nature Reserve, Heilongjiang, China | This paper |
|  | Yancheng National Wetland Nature Reserve for Rare Birds, Jiangsu, China | Bird Talker, 2018 |
|  | Izumi, Kagoshima, Japan | Crane Park Izumi, 2017 |
|  | Cheolwon, Gangwon, Korea | GBIF, 2017 |
|  | Yancheng National Wetland Nature Reserve for Rare Birds, Jiangsu, China | This paper |
|  | Ibaraki, Japan | Bird Fan, 2014 |
|  | Ibaraki, Japan | Bird Fan, 2014 |
| 2012-2013 | Izumi, Kagoshima, Japan | GBIF, 2017 |
|  | Yancheng National Wetland Nature Reserve for Rare Birds, Jiangsu, China | Cheng et al., 2014 |
|  | Chongming Dongtan Nature Reserve, Shanghai, China | Cheng et al., 2014 |
|  | Yancheng National Wetland Nature Reserve for Rare Birds, Jiangsu, China | This paper |
|  | Cheolwon, Gangwon, Korea | Kwnews, 2012 |
|  | Yancheng National Wetland Nature Reserve for Rare Birds, Jiangsu, China | Bird Talker, 2018 |
|  | Yancheng National Wetland Nature Reserve for Rare Birds, Jiangsu, China | Bird Talker, 2018 |
|  | Shanghai, China | Bosma, 2017 |
|  | Izumi, Kagoshima, Japan | Crane Park Izumi, 2017 |
|  | Izumi, Kagoshima, Japan | GBIF, 2017 |
|  | Izumi, Kagoshima, Japan | GBIF, 2017 |
|  | Izumi, Kagoshima, Japan | GBIF, 2017 |
|  | Izumi, Kagoshima, Japan | GBIF, 2017 |
|  | Izumi, Kagoshima, Japan | GBIF, 2017 |
|  | Izumi, Kagoshima, Japan | GBIF, 2017 |
|  | Izumi, Kagoshima, Japan | GBIF, 2017 |
|  | Izumi, Kagoshima, Japan | GBIF, 2017 |
|  | Guanzidong wetland, Liaoning, China | Cheng et al., 2014 |
|  | Lianyungang, Jiangsu, China | Bird Talker, 2018 |
|  | Sheyang, Yancheng, Jiangsu, China | Bird Talker, 2018 |
|  | Kagoshima, Japan | Bird Fan, 2014 |
|  | Kagoshima, Japan | Bird Fan, 2014 |
|  | Kagoshima, Japan | Bird Fan, 2014 |
| 2013-2014 | Izumi, Kagoshima, Japan | GBIF, 2017 |
|  | Izumi, Kagoshima, Japan | GBIF, 2017 |
|  | Izumi, Kagoshima, Japan | GBIF, 2017 |
|  | Izumi, Kagoshima, Japan | GBIF, 2017 |
|  | Izumi, Kagoshima, Japan | GBIF, 2017 |
|  | Izumi, Kagoshima, Japan | GBIF, 2017 |
|  | Yancheng National Wetland Nature Reserve for Rare Birds, Jiangsu, China | This paper |
|  | Miyun Reservoir, Beijing | Cheng et al., 2014 |
|  | Yancheng, Jiangsu, China | This paper |
|  | Yancheng National Wetland Nature Reserve for Rare Birds, Jiangsu, China | Bird Talker, 2018 |
|  | Izumi, Kagoshima, Japan | Crane Park Izumi, 2017 |
|  | Izumi, Kagoshima, Japan | GBIF, 2017 |
|  | Izumi, Kagoshima, Japan | GBIF, 2017 |
|  | Yancheng, Jiangsu, China | GBIF, 2017 |
|  | Izumi, Kagoshima, Japan | GBIF, 2017 |
|  | Izumi, Kagoshima, Japan | GBIF, 2017 |
|  | Kagoshima, Japan | This paper |
| 2014-2015 | Cheolwon, Gangwon, Korea | GBIF, 2017 |
|  | Izumi, Kagoshima, Japan | GBIF, 2017 |
|  | Yellow River Delta Nature Reserve, Shandong, China | Ifeng, 2014 |
|  | Tangshan, Hebei, China | Huanbohainews, 2014 |
|  | Zhalong National Nature Reserve, Heilongjiang, China | This paper |
|  | Kagoshima, Japan | Bird Fan, 2014 |
|  | Izumi, Kagoshima, Japan | Crane Park Izumi, 2017 |
|  | Yancheng National Wetland Nature Reserve for Rare Birds, Jiangsu, China | This paper |
| 2015-2016 | Izumi, Kagoshima, Japan | GBIF, 2017 |
|  | Izumi, Kagoshima, Japan | GBIF, 2017 |
|  | Izumi, Kagoshima, Japan | GBIF, 2017 |
|  | Izumi, Kagoshima, Japan | GBIF, 2017 |
|  | Caizi Lake, Anhui, China | This paper |
|  | Zhalong National Nature Reserve, Heilongjiang, China | This paper |
|  | Izumi, Kagoshima, Japan | Crane Park Izumi, 2017 |
|  | Chungcheongnam, Korea | GBIF, 2017 |
| 2016-2017 | Suncheon Bay Wetland, Suncheon, Jeonnam, Korea | Fun World, 2016 |
|  | Yancheng National Wetland Nature Reserve for Rare Birds, Jiangsu, China | Bird Report, 2016 |
|  | Yancheng National Wetland Nature Reserve for Rare Birds, Jiangsu, China | Bird Report, 2016 |
|  | Izumi, Kagoshima, Japan | Crane Park Izumi, 2017 |
|  | Suncheon Bay Wetland, Suncheon, Jeonnam, Korea | News1 |
| 2017-2018 | Izumi, Kagoshima, Japan | Crane Park Izumi, 2017 |

**REFERENCE**

Bird Fan. (2018) *Japanese Wild Birds Association*. Available at: <https://www.birdfan.net/pg/kind/ord08/fam0801/spe080103/> (accessed 2018).

Bird Report. (2016) *China birdwatching record center*. Available at: <http://www.birdreport.cn/Watch/RecordList4Field> (accessed 2018).

Bird Talker (2018) *China Bird Records Center*. Available at: http://www.szbird.org.cn/birdtalker.net/index.asp (accessed 2018).

Bosma (2017) *Chinese wild bird quick check (2.3.1) [Mobile application software]*. Available at: <http://itunes.apple.com> (accessed 2017).

Cheng, Y., Tang, L., Su, L., Zhou, H. & Ding, C. (2014) The distribution of the sandhill crane (*Grus canadensis*) in China. *Chinese Journal of Zoology*, **49**, 921-924.

Crane Park Izumi. (2017) *Number of crane transfers by year*. Available at: http://www.city.kagoshima-izumi.lg.jp/page/page_80092.html (accessed 2018).

Cai, Y., Tang, S., Yuan, X., Wang, J. & Ma, Z. (2011) Shanghai bird records and changes. *Journal of Fudan University(Natural Science)*, 334-343.

Fun World. (2016) *Sandhill crane*. Available at: <http://hudadag.tistory.com/861> (accessed 2018).

GBIF (2017) *Occurrence Download*. Available at: http://www.gbif.org/ (accessed 19 November 2017).

Huanbohainews (2014) *The crane species that are rare in China appeared along the beach of Tangshan (Photos)*. Available at: <http://tangshan.huanbohainews.com.cn/system/2014/10/24/011408047.shtml> (accessed 2018).

Ifeng (2010) *Rare birds found in many places in Heilongjiang, sandhill cranes were discovered for the first time in Yichun*. Available at: http://finance.ifeng.com/roll/20100527/2241185.shtml (accessed 2018).

Ifeng (2014) *Rare sandhill cranes reappeared in Dongying, which is really a rare and marvellous scene*. Available at: <http://sd.ifeng.com/news/chengshi/detail_2014_12/20/3315216_0.shtml> (accessed 2018).

Kuang, B., Xian, R. & Wang, Z. (1981) A new record of crane from China. *Zoological Systematics*, **2**, 99.

Kwnews (2012) *Capturing the internationally rare species 'sandhill cranes'*. Available at: http://www.kwnews.co.kr/nview.asp?s=501&aid=212112000103 (accessed 2018).

Liu, Z. & Ding, T. (1988) It was found that Canadian cranes wintering in Poyang Lake. *Chinese Journal of Wildlife*.

Ma, J. (2003) Sandhill cranes found in Zhalong Nature Reserve. *Chinese Journal of Wildlife*, 59.

Masatomi, H. (1972) Communal Wintering of a Sandhill Crane with Japanese Cranes in Hokkaido, Japan. *Biodiversity Heritage Library OAI Repository*, **84**, 250-260.

Naver Blog. (2006) *Looking for hooded crane and sandhill crane*. Available at: https://blog.naver.com/resetpkc/140022586126 (accessed 2018).

News1 (2017) *Suncheon News*. Available at: <http://news1.kr/articles/?3143764> (accessed 2018).

Qian, F. (2005) Survey on Current Status of Crane Protection in China. *Forest & Humankind*, 31-38.

USFWS (2018) *Sandhill Cranes*. Available at: https://www.fws.gov/birds/surveys-and-data/webless-migratory-game-birds/sandhill-cranes.php (accessed

Wang, X., Wu, X., Xin, H., Han, J. & Xu, J. (2007) Study on the Migration of Cranes in the Yellow River Delta Nature Reserve during the South Migration Period. *Shandong forestry science and technology*, 57-58+99.

Wang, Z. (2006) *Geographic distribution and in situ conservation of cranes in China*. Nanjing Normal University,

Yu, G., Wang, H., Sun, P. & Yang, P. (2011) A new record of bird from Jilin province: sandhill crane. *Journal of Northeast Normal University (Natural Science Edition)*, **40**, 159-160.
